# Removal Efficiency of Bottom Ash and Sand Mixtures as Filter Layers for Fine Particulate Matter

**DOI:** 10.3390/ma17112749

**Published:** 2024-06-05

**Authors:** Yunje Lee, Donghyun Lee, Hongkyoung Lee, Hyun-Seok Choe, Jae-Hyuk Kim, Yongjin Choi, Jaehun Ahn

**Affiliations:** 1 Department of Civil and Environmental Engineering, Pusan National University, Busan 46241, Republic of Korea; lee_yunje@pusan.ac.kr (Y.L.); choehs94@pusan.ac.kr (H.-S.C.); jaehyuk.kim@pusan.ac.kr (J.-H.K.); 2 Global Business Energy Infra Team, HanmiGlobal Co., Ltd., Seoul 06164, Republic of Korea; leedhyun@hanmiglobal.com; 3 Construction Division, NEODNC Co., Ltd., Busan 48106, Republic of Korea; adidars@naver.com; 4 Maseeh Department of Civil Architectural and Environmental Engineering Austin, The University of Texas at Austin, Austin, TX 78712, USA

**Keywords:** permeable pavement, bottom ash–sand mixture, silica sand, bottom ash, removal efficiency, filter layer

## Abstract

Permeable pavement is a technology that allows rainwater to infiltrate into the pavement. Permeable pavements not only help reduce surface runoff by allowing rainwater to infiltrate into the pavement, but also improve water quality with the filter layer that removes particulate matter pollutants. This study evaluated the particulate matter removal efficiency of bottom ash–sand mixtures as filter layers for removing fine (≤10 μm) or ultrafine (≤2.5 μm) particulate matter in the laboratory. Five filter media were tested: silica sand, bottom ash, and bottom ash–sand mixtures with 30:70, 50:50, and 70:30 ratios. The mixed filters exhibited more consistent and stable particulate matter removal efficiency over time than either the uniform sand or bottom ash filter. The 50:50 bottom ash–sand mixture demonstrated removal rates of 58.05% for 1.8 μm particles, 93.92% for 10 μm particles, and 92.45% for 60 μm particles. These findings highlight the potential of bottom ash–sand mixtures as effective filter media for removing PM10 road dust, although field validation with actual pavement systems is necessary.

## 1. Introduction

Urbanization has increased impervious surface areas, leading to groundwater depletion, elevated peak flows, and river water pollution [1,2]. One of the solutions to these problems is the use of permeable pavements [3,4]. Permeable pavements not only help reduce surface runoff by allowing rainwater to infiltrate into the pavement, but also improve water quality with the filter layer that removes particulate matter pollutants [5,6,7]. In the United States and Europe, where the management of non-point pollutants has become common, permeable pavement systems with permeable aggregates are widely used in efforts to reduce stormwater pollution loads and thereby improve river water quality [8,9].

While vehicle exhaust emissions have traditionally been considered the primary source of pollution on the road, stricter regulations have led to a significant decrease in particulate matter (PM) emitted from vehicle exhausts [10]. However, non-exhaust sources, such as tire and brake wear, remain a significant concern [11,12,13,14]. Harrison et al. [15] analyzed the composition of particles in the 0.9–11.5 μm size range collected on Marylebone Road in central London, and found that brake wear particles accounted for 55.3 ± 7.0%, tire wear particles for 10.7 ± 2.3%, and re-scattered dust for 38.1 ± 9.7% of the total mass. In Seoul metropolitan city, road re-scattered dust accounts for more than 20% of the source of PM10 (particulate matter ≤10 μm [16,17]) and PM2.5 (particulate matter ≤2.5 μm [16,17]) emissions. The re-scattered dust, especially fine contents like PM10 and PM2.5, causes respiratory symptoms [18,19].

Previous studies have shown that permeable pavements effectively mitigate particles less than 100 μm, with meaningful filtration performance [20,21,22,23,24]. Segismundo et al. [23] evaluated the filtration performance of sand filters for the sediment sizes ranging from 100 to 250 μm based on total suspended solids (TSS) removal efficiency. Nguyen et al. [22] evaluated the TSS removal efficiency of the sand filter for the smaller particles (≤60 μm). Kim et al. [24] analyzed the TSS removal efficiency of sand or bottom ash layers. García-Haba et al. [25] investigated the removal efficiency of permeable pavements for microplastic and tire wear particles smaller than ≤100 μm. However, studies on the filtration performance for very fine particulate matter (i.e., PM10 and PM2.5) are insufficient, despite their known harmful effects on human health.

This study aims to experimentally evaluate the filtration performance of bottom ash, silica sand, and bottom ash–sand mixtures—potential materials for filter layers in permeable pavement systems—for fine particulate matter based on their removal efficiency. The removal efficiency of the bottom ash–silica sand mixture has not been tested previously, to the best of the authors’ knowledge, for removing fine particulate matter like PM10 and PM2.5. First, the removal efficiencies of bottom ash–sand mixtures with different mixture proportions for 60 μm particles are tested. Based on the test results, a filter with the optimum blend ratio is selected. Then, the removal efficiency of the selected filter for fine particles (10 μm and 1.8 μm, which fall in the definition of PM10 and PM2.5) is tested.

## 2. Materials and Methods

### 2.1. Filter Materials

**Figure 1:**
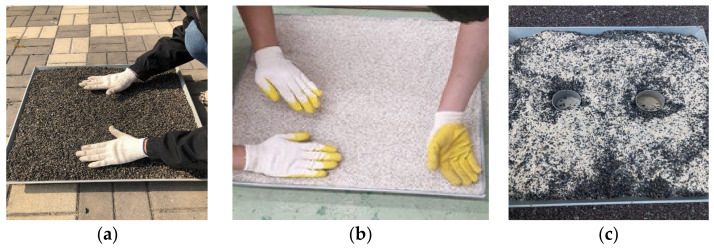
Filter materials: (**a**) Bottom ash, (**b**) silica sand, (**c**) bottom ash–sand mixture. The size of the pan containing the materials is 60 × 60 × 7.5 cm.

Figure 1 shows the filter materials tested in this study: bottom ash, silica sand, and bottom ash–sand mixtures. Bottom ash is a type of coal ash that is the residue left after burning coal in a boiler in a thermal power plant in the process of thermal power generation [26]. In general, 20–30% of the coal ash is melted by the high temperature combustion heat, condensed, and then, falls into the clinker hopper under the boiler, which is called bottom ash [27]. The bottom ash used in this study (Figure 1a) was sourced from a thermal power plant in South Korea. For the sand material (Figure 1b), particle size type No. 5 silica sand was purchased from a manufacturer and used. For the mixed material (Figure 1c), three different blend ratios were prepared: 30:70, 50:50, and 70:30 bottom ash-to-silica sand by weight. These bottom ash–sand mixtures were for investigating the effects of varying proportions on filtration performance. Prior to testing, the filter materials were carefully washed with running water to minimize extraction of fines from the filter itself and dried at 110 °C for 48 h.

**Figure 2:**
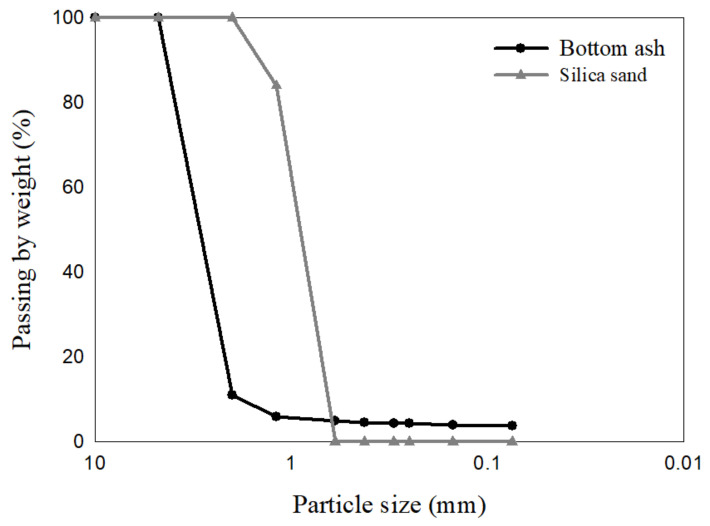
Particle size distribution of bottom ash and silica sand.

The particle size distributions of bottom ash and silica sand were analyzed using standard sieves Nos. 4, 10, 16, 30, 40, 50, 60, 100, and 200 [28]. Figure 2 shows the sieve analysis results. For the bottom ash, the coefficient of uniformity was evaluated to be 1.85 and the coefficient of curvature 0.89. In the case of the silica sand, the coefficient of uniformity was 1.49 and the coefficient of curvature was 0.92.

### 2.2. Unit Weight of Filter Materials

In order to determine the degree of compaction of the filter specimens during vibration compaction, the unit weights of the filter materials over the compaction time were evaluated experimentally. The test setup consisted of a square pan (60 × 60 × 7.5 cm) where the filter materials were compacted, and two cylindrical sample cans (7.5 cm diameter, 5 cm height) placed inside the pan for measuring the local unit weight during the compaction. The test procedure was as follows: (1) place two sample cans inside a square pan; (2) spread the filter material in the square pan, filling the sample cans, and flatten the surface; (3) compact the filter material in the pan using a vibratory compactor for a specified time; and (4) after compaction, carefully retrieve the sample cans from the pan, and measure the weight of the compacted filter material within the sample cans.

The unit weights of three samples—silica sand, bottom ash, and bottom ash–sand mixture (50:50 weight ratio)—were analyzed. After 25 s of vibration compaction, the unit weights of the silica sand, bottom ash, and bottom ash–sand mixture reached 14.46 kN/m^3^, 7.49 kN/m^3^, and 12.65 kN/m^3^, respectively. Further vibration did not result in additional increases in unit weight. Accordingly, the unit weights at 25 s of vibrations were set as baselines in the experimental program.

### 2.3. Permeability Coefficient

**Figure 3:**
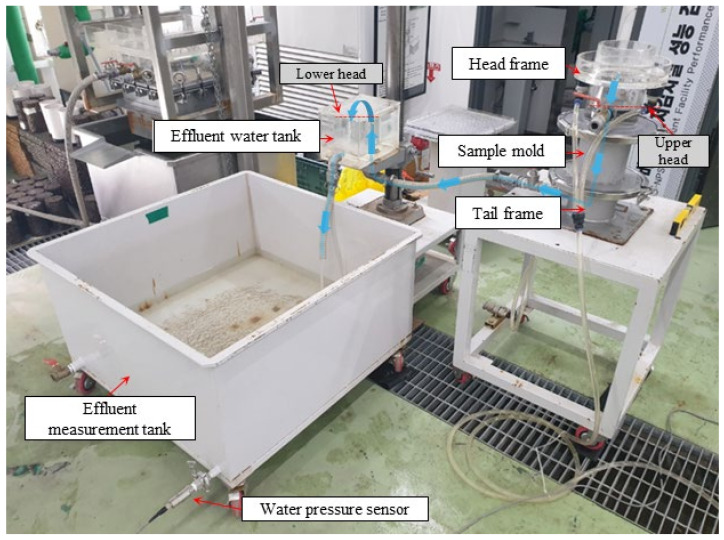
Testing equipment for permeability coefficient [29]. It consists of a head frame, sample mold, tail frame, effluent tank, and measurement tank. The blue line and the arrows show the water flow direction during the test.

The test equipment, developed by Ahn et al. [29], was used to evaluate the permeability coefficients of the filter media (Figure 3). The test equipment consists of head frame, sample mold (15 (diameter) × 10 cm), tail frame, effluent tank, and measurement tank. The level of the effluent tank can be adjusted to change the lower water head, and thus, the hydraulic gradient. To install the test sample in the sample mold, a geotextile is first installed at the bottom, and then, the filter sample is compacted over it with the specified unit weight. The sample mold is installed on the tail frame, fixed, and sealed with a clamp, and then, a head frame is installed on the sample mold in the same way. A water pressure sensor is installed on the measuring tank to measure the flow rate. 

During the test, water was introduced to the head frame. The water then infiltrated within the sample mold, and flowed out through the tail frame, effluent tank, and measurement tank, subsequently (see the blue line with arrows in Figure 3). The water head difference between the upper head at the head frame and the lower head at the effluent tank (Δh) was fixed to maintain a constant head (see the dashed red lines in Figure 3). A pressure sensor installed on the measurement tank continuously monitored the change in water level within the tank. The data obtained from the pressure sensor were used to calculate the discharge velocity of water flowing through the sample. 

Once the test setup was complete, the following procedure was used to carry out the tests: (1) adjust the level of the effluent tank to set the specified hydraulic gradient (the hydraulic gradient i was set to 1.0 during the tests); (2) allow water to enter the headframe and maintain a constant water level; (3) record the flow rate through the water pressure sensor in the measurement tank; (4) calculate the permeability coefficient k from the discharge velocity v and hydraulic gradient i in Equation (1).

(1) $$v\left(\mathrm{mm}/s\right)=ki=k\frac{\Delta h}{L}$$

Here, L is the height of the sample, which is 10 cm; Δh, the head difference between the upper head and lower head (see the red dashed lines in Figure 3), is 10 cm, resulting in i=1.0. The test was performed in an indoor laboratory at room temperature minimizing the effect of temperature on the measurements. More details about the test equipment and the procedures can be found in Ahn et al. [29].

### 2.4. Removal Efficiency for 60 μm Particles

This study first tested the particulate matter removal efficiency of bottom ash–sand filters for large particulate matter. Particulate matter that satisfied the PM60 specification [16,17] (particle diameter of 60 μm or less) was prepared. This falls within the Environmental Protection Agency (EPA) standard definition of silt and clay (<63 μm) [30]. Contaminated water containing 60 μm particulate matter was prepared with a target concentration ranging from 100 to 150 mg/L, which is similar to the recommended values from The Ministry of Environment [31] of South Korea. The range was used rather than a specific value due to some inherent variation during the test, despite efforts to maintain a consistent concentration. Five bottom ash–sand filters with varying material composition ratios, shown in Table 1, were used to evaluate the effect of filter composition on particulate removal efficiency.

**Figure 4:**
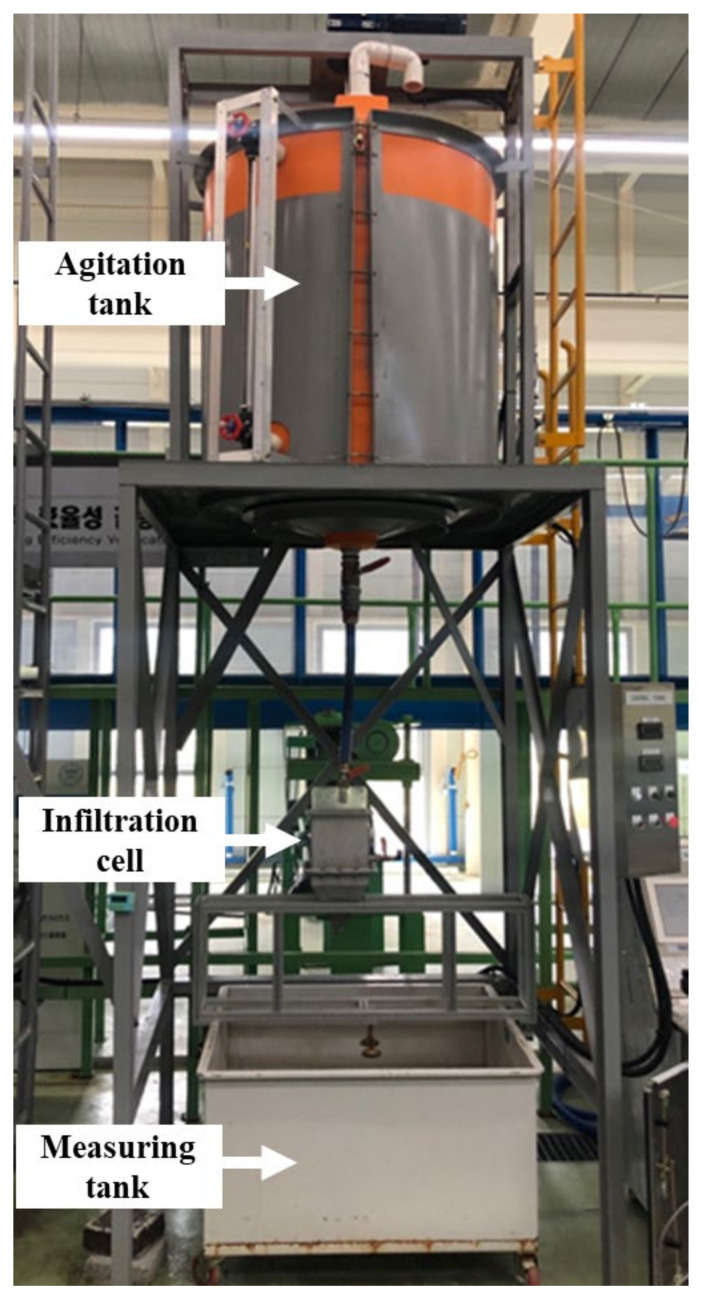
Test equipment for particulate matter removal efficiency for 60 μm particles. The water infiltrates from the top of the specimen.

Figure 4 shows the test equipment for evaluating the particulate matter removal efficiency of filters for 60 μm particles. It consists of an agitation tank, an infiltration cell, and an effluent tank. The maximum capacity of the agitation tank is 1000 L, and the amount of contaminated water in the tank can be viewed through a side window. The stirring speed can be adjusted from the control panel to a maximum of 400 rpm. The agitation tank supplies the contaminated water to the 20 × 20 × 12 cm infiltration cell, where the filter sample is placed. This square-shaped cell geometry is designed to test filter samples installed in actual pavement systems, including interlocking permeable block pavements. The filtered water flows into the effluent tank. The outflow water is measured at the outlet of the infiltration cell for measuring concentration.

The details of the experimental procedure to evaluate the particulate removal efficiency are as follows: (1) introduce clean water into the infiltration cell and collect inflow and outflow water samples at the inlet and outlet of the cell after 1, 10, and 20 min. (this procedure is to evaluate the amount of fine contents from the filter sample itself); (2) place the water and particulates in the agitation tank and stir at 270 rpm for 30 min; (3) add contaminated water to the infiltration cell and collect water samples at the inlet and outlet of the cell after 1, 10, 20, 30, 40, and 50 min for recording removal efficiency variation with time; (4) evaluate the concentrations of particulate matter of the inflow and outflow water samples collected according to the ASTM standard [32]; (5) calculate the removal efficiency, *RE*, using Equation (2), where *C_in_* and *C_out_* are the concentrations of the inflow and outflow water.

(2) $$RE\left(\%\right)=\frac{C_{in}-C_{out}}{C_{in}}\times 100$$

### 2.5. Removal Efficiency for 1.8 μm, 10 μm, and 60 μm Particles

Fine particulate matter has a particle diameter of 10 μm or less (PM10), and ultrafine particulate matter has a particle diameter of 2.5 μm or less (PM2.5). The particulate matter removal efficiency of the A50 filter sample was tested for 1.8 μm particles, which corresponds to PM2.5, and 10 μm particles, which corresponds to PM10, in addition to 60 μm particles. The contaminated water with each particulate matter was prepared with a target concentration of ~200 mg/L.

**Figure 5:**
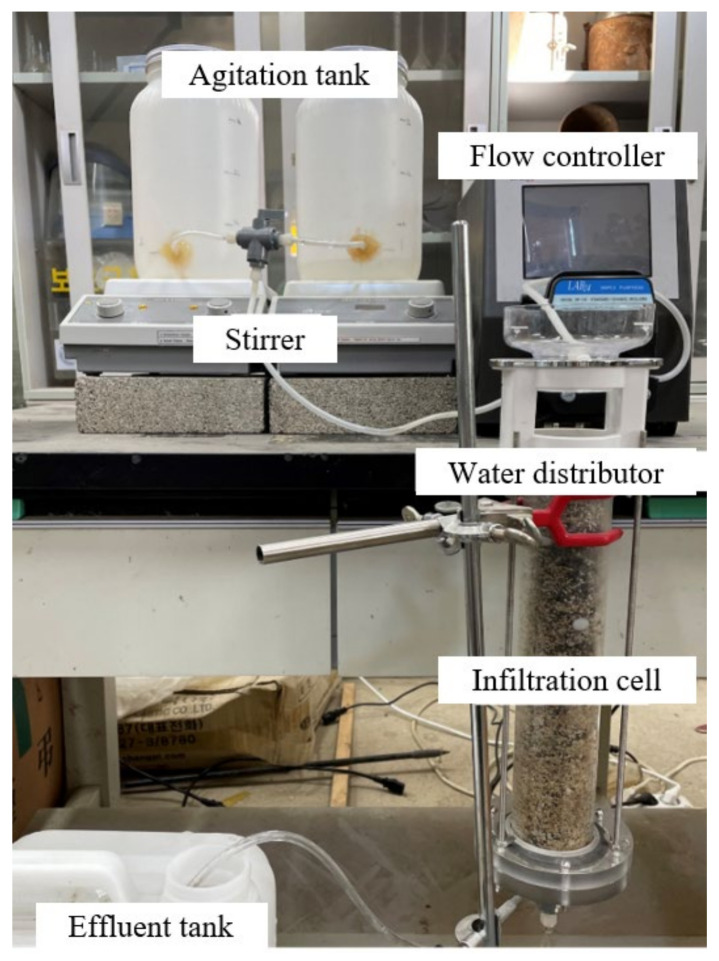
Test equipment for particulate matter removal efficiency for 1.8 μm, 10 μm, and 60 μm particles. The water infiltrates through the specimen from the top of the specimen.

Figure 5 shows the test equipment for evaluating the particulate matter removal efficiency of filters for fine particles and 60 μm particles. The design of this equipment was based on the original work of Won et al. [33]. It consists of agitation tank, flow controller, water distributor, infiltration cell, and effluent tank. Clean water and particulate matter are mixed in the agitation tank, which has magnetic stirrers, and delivered to the infiltration cell by the flow controller. The water distributor has a spinning bowl with holes to distribute the water evenly over the sample in the infiltration cell. The size of the infiltration cell is 5 (diameter) × 30 cm.

The details of the experimental procedure for evaluating the removal efficiency of particulate matter are as follows: (1) introduce clean water into the infiltration cell at a rate of 150 mL/min for 20 min; (2) place the water and particulate matter in the agitation tank and stir at 500 rpm for 20 min; (3) add contaminated water to the infiltration cell at a rate of 150 mL/min and collect water samples at the inlet and outlet of the cell after 1, 10, 20, 30, 40, 50, and 60 min for recording removal efficiency variation with time; (4) evaluate the particulate matter concentrations of the inflow and outflow water samples collected according to the ASTM standard [32]; (5) calculate the removal efficiency, *RE*, using Equation 2, where *C_in_* and *C_out_* are the particulate matter concentrations of the inflow and outflow water.

## 3. Results and Discussion

### 3.1. Permeability Coefficient

Permeability coefficient tests were conducted on five different filter specimens with varying proportions of bottom ash and sand, as shown in Table 1 (see Section 2.4). The two-digit number in the specimen ID represents the proportion of bottom ash in the bottom ash–sand filter mixture by weight. Sample A00 refers to a uniform sample of 0% bottom ash (or 100% silica sand), and the unit weight is 14.46 kN/m^3^, as determined by the vibration compaction test described in Section 2.2. Sample A100 is 100% bottom ash, and the unit weight is 7.49 kN/m^3^. Sample A50 is 50% bottom ash (and therefore 50% sand), and the unit weight is 12.65 kN/m^3^. Samples A30 and A70 have the same unit weight as A50 (12.65 kN/m^3^) but different proportions of the mixture, consisting of 30% and 70% bottom ash, respectively. Knowing the dry unit weights of silica and bottom ash beforehand (Table 1), the weight of the specimen required to match the cell volume corresponding to 12.65 kN/m^3^ was calculated. The specimens were then compacted for different durations until the target unit weight was consistently achieved.

Table 2 summarizes the permeability coefficient test results for five mixed bottom ash and sand filter samples, tested based on Section 2.3. Each sample was tested three times, and the average values were reported with their standard deviations. As stated in Table 1, samples A00, A50, and A100 were subjected to the same duration (25 s) of vibratory compaction, resulting in different unit weights (see Section 2.2). On the other hand, samples A30, A50, and A70 were compacted with the same unit weight (the same criteria for unit weight in the field). When comparing the permeability coefficient for the two uniform samples A00 and A100, A100 (8.75 mm/s) showed a higher permeability coefficient than A00 (5.79 mm/s). All three mixed samples with different proportions, A30, A50, and A70, had a lower permeability coefficient than the uniform samples, A00 and A100. Among the mixed samples, the ones with higher bottom ash content tended to have a lower permeability coefficient (4.26 mm/s) with no significant difference among the samples.

### 3.2. Particulate Matter Removal Efficiency (PM60)

Before injecting the contaminated water into the infiltration cell, clean water was injected into the infiltration cell for 20 min to check the amount of particulate matter emitted from the filter itself. It was found that the particulate matter concentration of water at the outlet of the cell was only 0–5 mg/L, which can be considered an error range in the particulate matter concentration of the filtered water.

**Figure 6:**
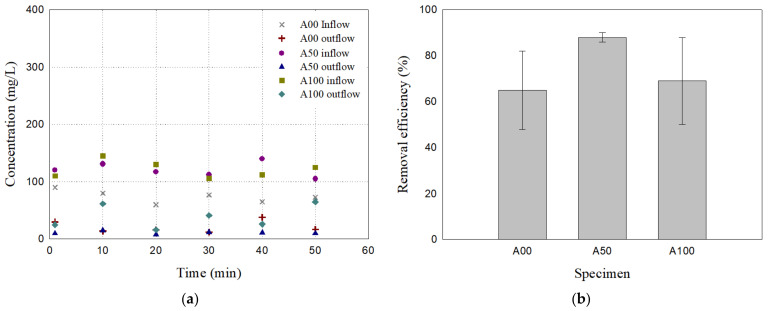
The particle removal test results of the filter specimens A00, A50, and A100 for 60 μm particles: (**a**) Inflow and outflow concentration; (**b**) removal efficiency averaged. The error bars represent the minimum and maximum values of the measurements.

Figure 6 shows the particle removal test results of the filter specimens A00, A50, and A100 for 60 μm particles. The uniform silica sand sample (A00) and the uniform bottom ash sample (A100) exhibited a similar average removal efficiency of 70.45% and 68.92%, respectively. In comparison, the mixed sample (A50) demonstrated a higher average removal efficiency of 89.06%, outperforming the uniform samples. In addition, the removal efficiency of the uniform samples (A00 and A100) varied greatly with time, whereas the mixed sample (A50) showed a consistent and stable performance, which can be also observed in their standard deviations (see Table 3). Considering that the recommended total suspended solids removal rate is generally 80% [34], the bottom ash–sand mixture filter A50 may be a suitable option for practical applications.

**Figure 7:**
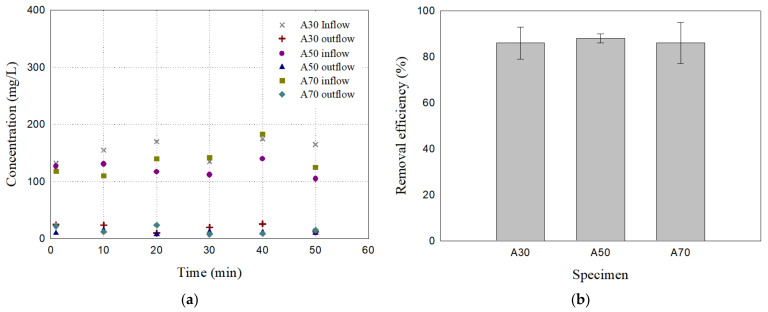
The particle removal test results of the filter specimens A30, A50, and A70 for 60 μm particles: (**a**) Inflow and outflow concentration; (**b**) removal efficiency. The error bars represent the minimum and maximum values of the measurements.

Samples A30 and A70, which have the same unit weight as A50, were prepared and tested (see Table 1). Figure 7 presents the particle removal test results of the filter specimens A30, A50, and A70 for 60 μm particles. A30 and A70 exhibited an average removal efficiency of 87.30% and 87.60%, respectively, which are comparable to that of A50 at 89.06%. The removal efficiencies of the mixed samples showed more consistent results than uniform samples (Table 3). The removal efficiency results in Figure 6b and Figure 7b indicate that all three mixed filters (A30, A50, and A70), which have the same unit weight but different proportions of mixture, provide better removal efficiencies than uniform filters (A00 and A100) for 60 μm particles.

### 3.3. Particulate Matter Removal Efficiency (PM10 and PM2.5)

**Figure 8:**
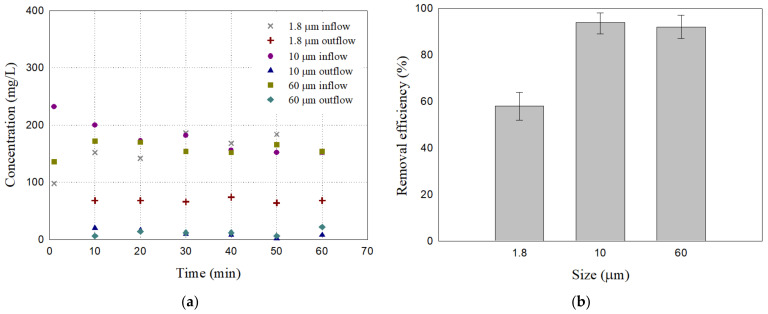
The particle removal test results of the filter specimen A50 for 1.8 μm, 10 μm, and 60 μm particles: (**a**) Inflow and outflow concentration; (**b**) removal efficiency averaged. The error bars represent the minimum and maximum values of the measurements.

Figure 8 shows the removal efficiencies of the filter specimen A50 for 1.8 μm, 10 μm, and 60 μm particles tested with the setup in Figure 5. The A50 mixed filter presented an average removal efficiency of 92.45% for 60 μm particles and 93.92% for 10 μm particles. The removal efficiency of these samples remained consistent over the measurement period, with slight variation (see Table 4). The removal efficiency for 1.8 μm particles was, on the other hands, 58.05% which is lower than those for 10 μm, and 60 μm particles. It appears that the size and arrangement of the pore in the A50 filter are not proper to filter the particles as fine as 1.8 μm. Consequently, the bottom ash and sand mixture successfully filtered 10 μm particles but not 1.8 μm ones. Further research is needed to validate the removal efficiencies of traditional granular filters for very fine particulate matters, and to develop new filter materials.

## 4. Conclusions

This study experimentally evaluated the permeability coefficients and particle removal efficiencies of bottom ash–sand filter materials. Five filter media were tested: silica sand (A00), bottom ash (A100), and bottom ash–sand mixtures with ratios of 30:70 (A30), 50:50 (A50), and 70:30 (A70). The following conclusions are drawn from the results.

- Permeability coefficients: Among A00, A100, and A50, which are compacted for the same duration by vibration, resulting in different unit weights, the mixed filter (A50) had a lower permeability coefficient than the uniform filters (A00 and A100). Among A30, A50, and A70, which are designed to have the same unit weight, the one with higher bottom ash content (A70) tended to have a lower permeability coefficient than the others.
- Particle removal efficiency for 60 μm particulates: Among the uniform filters (A00 and A100) and the mixed filter (A50), A50 showed a higher removal efficiency of 89.06% compared to 70.45% and 68.92% for A00 and A100, respectively. The other mixed filters, A30 and A70, showed a removal efficiency of 87.30% and 87.60%, respectively, which were comparable to that of A50. The mixed filters exhibited more consistent and stable removal efficiency over time compared to the uniform filters.
- Particle removal efficiency of A50 filters for different particles: A50 had removal efficiencies of 58.05%, 93.92%, and 92.45% for 1.8 μm, 10 μm, and 60 μm particles, respectively. These findings highlight the potential of bottom ash–sand mixtures as effective filter media even for PM10 road dust. The removal efficiency for 1.8 μm was not as good, but the A50 filter still screens out a considerable amount of the particles smaller than PM2.5. Further research may be required for the removal of finer particulate matter.

This study addresses the results obtained from the controlled laboratory experiments, but may not fully represent the performance of filter media in the field. For example, the removal efficiency of the filter may change over time due to clogging in the field. However, the emphasis of this study is on the comparison of the removal efficiencies of various filters for various particulate matter over about a one-hour duration. Field demonstration experiments are necessary to validate the bottom ash–sand filters for practical applications.

## Figures and Tables

**Table 1:** Material composition ratios of bottom ash–silica sand filters and their properties, used for testing the particulate removal efficiency.

Case	Specimen ID	Mixture Proportion (%)		Dry Unit Weight (kN/m^3^)
		Bottom Ash	Silica Sand	
The same compaction time (i.e., 25 s) ^1^	A00	0	100	14.46
	A50 ^3^	50	50	12.65
	A100	100	0	7.49
The same unit weight ^2^	A30	30	70	12.65
	A50 ^3^	50	50	
	A70	70	30	

^1^ Unit weight did not increase after 25 s with additional vibratory compaction. ^2^ The same unit weight was targeted. Knowing the dry unit weights of silica and bottom ash beforehand, the weight of the specimen required to match the cell volume corresponding to 12.65 kN/m^3^ was calculated. The specimens were then compacted for different durations until the target unit weight was consistently achieved. ^3^ The same specimen, but reiterated to better distinguish the cases.

**Table 2:** Permeability coefficients of the filter specimens.

Case	Specimen ID	Permeability Coefficient (mm/s)	Standard Deviation (mm/s)
The same compaction time	A00	5.79	0.073
	A50 ^1^	4.85	0.053
	A100	8.75	0.104
The same unit weight	A30	5.23	0.131
	A50 ^1^	4.85	0.053
	A70	4.26	0.033

^1^ The same specimen, but reiterated to better distinguish the cases.

**Table 3:** The standard deviations of the removal efficiencies of the test specimens.

Specimen ID	Standard Deviation (%)
A00	14
A30	5
A50	2
A70	5
A100	14

**Table 4:** The standard deviations of the removal efficiency of A50.

Particulate Matter	Standard Deviation (%)
1.8 μm	5
10 μm	3
60 μm	4

## Data Availability

Data are contained within the article.
